# Managing Medically Severe Self-Injury in Borderline Personality Disorder and Autism: A Case Report of Self-Induced Toe Gangrene

**DOI:** 10.7759/cureus.92509

**Published:** 2025-09-17

**Authors:** Khaled Alharmoodi, Dilshana Bapakunhi, Achsa Andrews, Hani Akasheh, Ahmed Shoka

**Affiliations:** 1 Psychiatry, Essex Partnership University NHS Foundation Trust, Colchester, GBR

**Keywords:** autism spectrum disorder (asd), borderline personality, borderline personality disorder, gangrene, ligature, self-harm

## Abstract

Medically severe self-injury (MSSI) in individuals with emotionally unstable personality disorder (EUPD) and co-occurring autism spectrum disorder (ASD) can be life- or limb-threatening, requiring urgent multidisciplinary intervention. We report a 31-year-old woman who developed self-induced toe gangrene following prolonged ligature application. Despite systemic stability, conservative management was pursued due to preserved vascular integrity, while psychiatric risk necessitated detention under the Mental Health Act. This case underscores the complex clinical, ethical, and legal challenges in balancing patient autonomy with safety, highlighting the importance of individualized, coordinated care across psychiatric, medical, and surgical teams.

## Introduction

Medically severe self-injury (MSSI) presents substantial clinical challenges, often resulting in chronic wounds, impaired healing, and heightened risk of infection. In the most severe cases, these injuries can progress to tissue necrosis or systemic complications. Although self-harm is relatively common among psychiatric populations, the subset of individuals whose behaviours cause permanent tissue damage or necessitate urgent medical intervention is considerably smaller yet clinically significant [1,2]. MSSI often occurs in the context of comorbid psychiatric disorders, including but not limited to emotionally unstable personality disorder (EUPD), autism spectrum disorder (ASD), depression, anxiety disorders, and psychotic disorders. Across these conditions, deficits in emotional regulation, impulse control, social cognition, and coping strategies can increase vulnerability to MSSI [3-5].

EUPD, characterized by pervasive affective instability, impulsivity, and recurrent self-harming behaviours, often serves as a maladaptive coping mechanism for managing overwhelming emotional states, perceived rejection, or interpersonal stress. Recent longitudinal research underscores the critical role of emotion dysregulation in these behaviours, suggesting that early intervention can lead to significant improvements in emotional regulation capacity in adulthood [4]. Although most acts are superficial, a minority escalate to medically severe behaviours, such as ischemic injury, ingestion of foreign objects, or self-inflicted burns, which pose substantial physical health risks [1].

ASD, a neurodevelopmental condition affecting social communication and behaviour, is associated with elevated rates of self-injury. Repetitive or body-focused behaviours, sensory processing difficulties, and challenges in emotional regulation may contribute to the persistence of self-harming actions [3,6].

The co-occurrence of EUPD and ASD may amplify the risk for MSSI, as impulsive, affect-driven behaviours intersect with rigid, perseverative tendencies, sensory sensitivities, and atypical pain perception [3,4]. Such complex presentations require careful risk assessment and a multidisciplinary approach, integrating psychiatric, surgical, and medical expertise to optimize outcomes and minimize iatrogenic harm [1].

This case report describes a 31-year-old woman with EUPD and ASD who developed self-induced toe gangrene following a relationship breakdown, demonstrating how this diagnostic combination can precipitate medically severe self-harm and underscoring the need for multidisciplinary management at the interface of psychiatry and medicine.

## Case presentation

A 31-year-old Caucasian woman with a 10-year history of EUPD (borderline subtype), characterized by affective instability and recurrent self-harming behaviours, and ASD requiring minimal support in daily functioning, was admitted to a psychiatric inpatient unit following a marked escalation in self-harm behaviours.

She had a long-standing history of poor community stability, including frequent psychiatric hospitalizations, difficulty maintaining employment, and disrupted interpersonal relationships. Over this period, she engaged in recurrent and severe self-injurious behaviours, including cutting, ingestion of foreign objects, and deliberate thermal burns, often requiring urgent medical attention.

A recent separation from her long-term partner precipitated an acute deterioration in her mental state. She described the breakup as highly triggering, during which she realized her partner had been emotionally abusive. This discovery reactivated traumatic memories of longstanding maternal abuse, resulting in profound emotional dysregulation, feelings of abandonment, and internalized shame.

**Figure 1:**
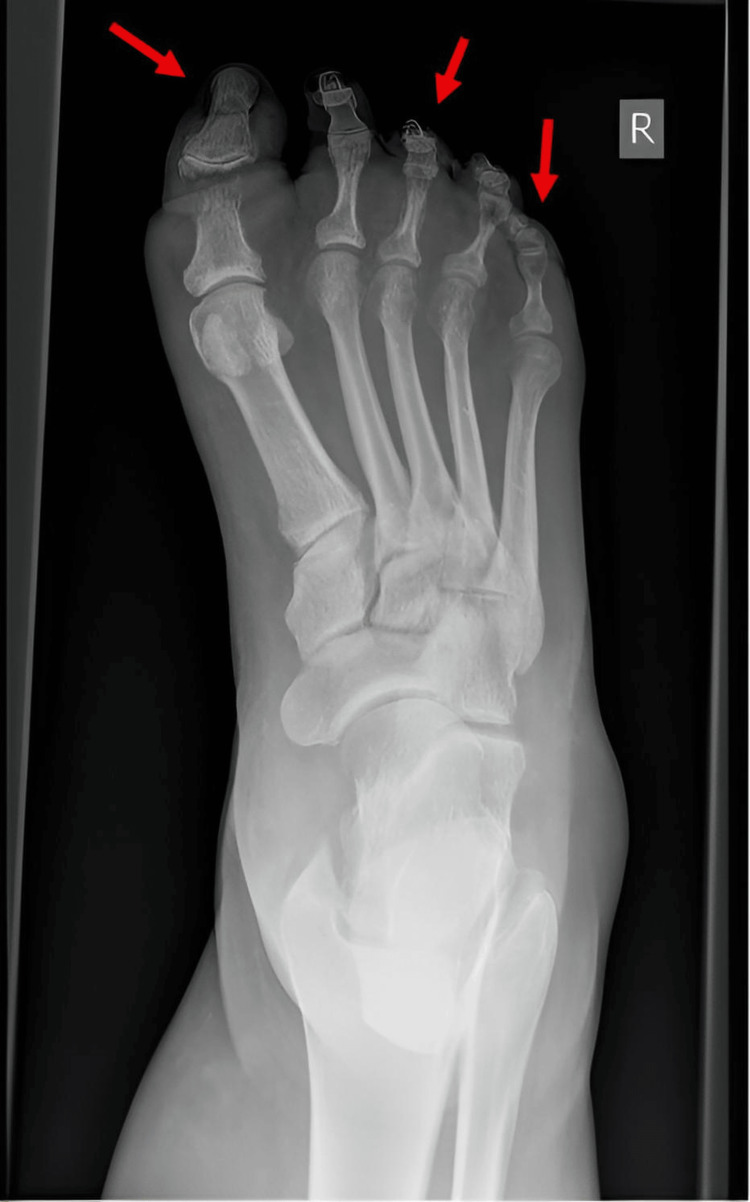
Initial foot radiograph showing osteomyelitis of the great toe with soft tissue gas and periosteal changes in the third and fifth toes.

Shortly after the relationship breakdown, she tied rubber bands tightly around each of her toes on the right foot for nine days. On presentation to the emergency department (ED), all toes were visibly gangrenous (Figure 1). She was systemically stable with preserved peripheral perfusion and normal inflammatory markers. A CT angiography of the right foot was performed and was normal, confirming intact arterial flow to the affected toes. Initial management included intravenous antibiotics (cefuroxime 1.5 g every eight hours) and analgesia (paracetamol 1 g orally every six hours and morphine 5 mg IV as needed), along with assessment by vascular and orthopaedic teams. She was then discharged on oral antibiotics (amoxicillin-clavulanate 875/125 mg twice daily for seven days) and analgesia as above before transfer back to the psychiatric unit.

**Figure 2:**
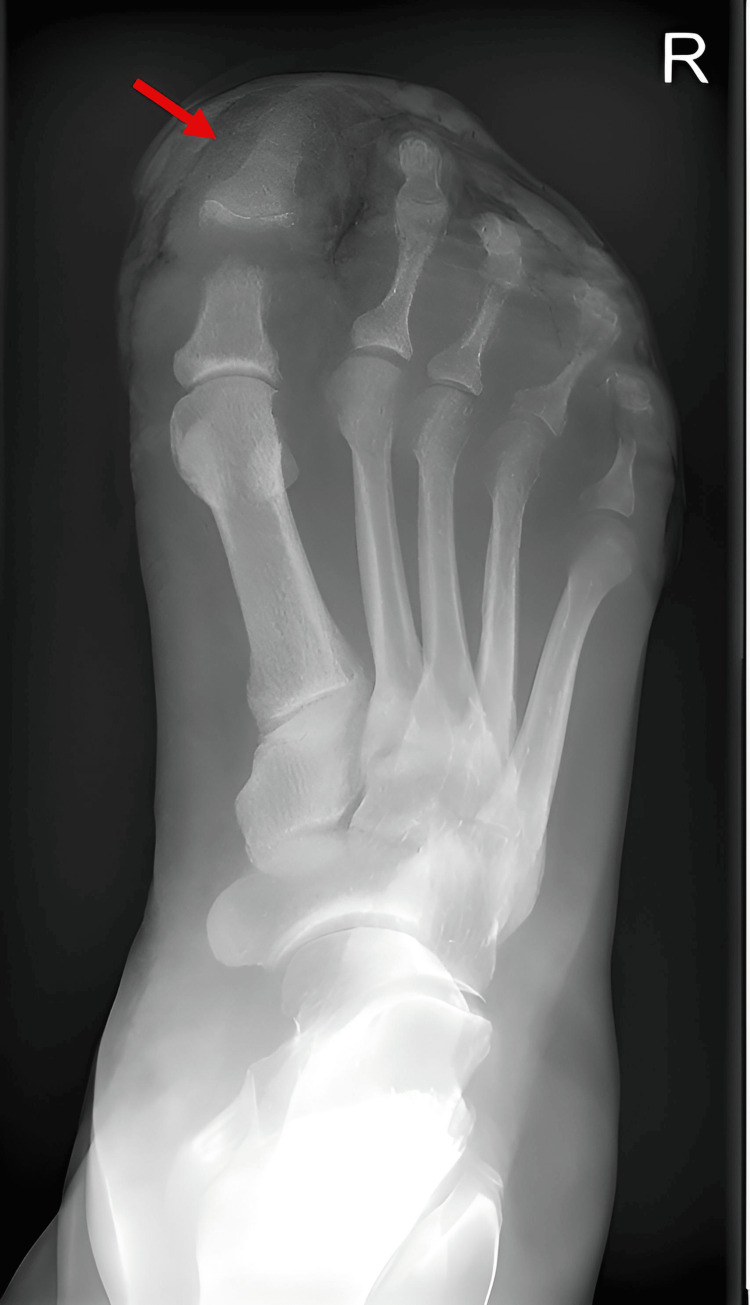
Follow-up radiograph two weeks later demonstrating progression of osteomyelitis in the great toe, with unchanged findings in the third and fifth toes.

During her admission, the patient refused to comply with antibiotics despite multiple attempts to persuade her. She expressed distress that surgical amputation had not been performed and continued to manipulate the affected toes, deliberately worsening tissue ischemia. Increasing pain prompted a second transfer to the ED, where surgical teams reassessed her (Figure 2). Given her normal vascular status confirmed by CT angiography, absence of diabetes mellitus (DM), and systemic stability, a conservative, non-surgical approach was adopted. The toes were expected to undergo auto-amputation through natural demarcation of necrotic tissue.

Psychiatric management included intensive dialectical behaviour therapy (DBT) techniques, though she had a history of limited response. Her medication regimen included duloxetine 120 mg orally once daily for depressive symptoms and anxiety, carbamazepine 200 mg orally three times daily for mood stabilization and impulse control, and nortriptyline 20 mg orally at bedtime, prescribed by the pain management team for sleep support and neuropathic pain. She had previously trialled multiple antipsychotics and antidepressants, which provided minimal clinical benefit. Despite these interventions, she continued to engage in high-risk self-harm with minimal warning. Due to escalating risk and concerns regarding her safety, following worsening of her physical condition and continued high-risk self-harm, she was deemed to lack capacity to make treatment decisions, and detention under the Mental Health Act was recommended. She was initially detained under Section 2 for assessment and subsequently under Section 3 for ongoing treatment. She was ultimately transferred to another hospital, with no further follow-up information available.

## Discussion

EUPD and ASD in severe self-harm

This case illustrates how the co-occurrence of EUPD and ASD can amplify the risk of medically severe self-harm. While self-injury in EUPD is often superficial, the patient’s prolonged and repeated application of constrictive rubber bands, ultimately resulting in toe gangrene, represents an uncommon and extreme presentation. The combination of impulsive, affect-driven behaviours characteristic of EUPD with rigid, perseverative tendencies and atypical sensory processing associated with ASD may have contributed to the persistence and escalation of harm [3,5,6].

Notably, the patient’s behaviour appears to reflect both emotional dysregulation and potential sensory modulation, suggesting that ASD-related factors may reinforce high-risk self-injury beyond the mechanisms typically observed in EUPD alone. This underscores the importance of assessing not only affective and impulsive tendencies but also repetitive behaviours, sensory sensitivities, and atypical pain perception when evaluating risk in individuals with dual diagnoses [3,5,6].

From a clinical perspective, cases such as this highlight the need for tailored, multidisciplinary interventions. Management should integrate psychiatric strategies targeting emotional regulation and behavioural control with close medical monitoring, particularly when self-injury carries a risk of severe tissue damage or systemic complications. Awareness of how EUPD and ASD can interact to amplify risk can inform proactive prevention strategies, individualized risk assessments, and coordinated care plans [1,4,5].

Self-induced gangrene in medically severe self-harm

Self-induced gangrene is a rare and severe manifestation of self-harm, reflecting the intersection of psychiatric vulnerability and serious physical injury [1]. In this case, the patient’s prolonged use of tight rubber bands caused ischemia and necrosis, further complicated by continued manipulation of the affected area and refusal of antibiotics [7-9]. Given her systemic stability, preserved peripheral perfusion, and absence of diabetes, a conservative, non-surgical approach was appropriate, allowing natural demarcation of necrotic tissue while minimizing unnecessary loss of viable tissue [7-9].

Careful observation and close monitoring were essential to ensure that infection did not develop, pain was adequately managed, and tissue changes were detected promptly. This case, therefore, illustrates how high-risk self-harm can require a delicate balance between preserving viable tissue and implementing proactive medical management, emphasizing vigilant monitoring, infection prevention, and pain control to optimize outcomes [7-9].

Effective multidisciplinary collaboration between psychiatric, surgical, and medical teams was critical, addressing both the underlying psychiatric drivers of self-harm and the resulting physical injuries [1,9].

Ethical and risk management considerations

In this case, the patient’s persistent self-harm and refusal of antibiotics presented a significant ethical dilemma, requiring a careful balance between respecting autonomy and ensuring beneficence. Following worsening of her physical condition and ongoing high-risk self-injury, she was deemed to lack the capacity to make informed treatment decisions, highlighting the importance of individualized capacity assessment, particularly in individuals with EUPD and comorbid ASD, where affective instability, impulsivity, and communication difficulties may further compromise decision-making [4].

Detention under the Mental Health Act (initially Section 2 for assessment and subsequently Section 3 for ongoing treatment) was implemented to ensure both psychiatric and medical interventions could be delivered safely while safeguarding her rights [10,11]. This approach underscores the necessity of integrating ethical reasoning, formal capacity assessment, and legal frameworks when managing severe self-harm, particularly in patients with complex comorbidities.

Furthermore, this case demonstrates how tailored risk management strategies and close interdisciplinary collaboration across psychiatric, surgical, and medical teams are essential to address both the underlying psychiatric drivers of self-harm and the resultant physical injuries [10].

## Conclusions

MSSI, including self-induced gangrene, represents a complex clinical challenge, particularly in the context of co-occurring EUPD and ASD. Management requires coordinated, multidisciplinary care integrating psychiatric, medical, and surgical expertise to address both physical and psychological needs. Ethical and legal frameworks, including capacity assessment and the application of the Mental Health Act, are essential when patients refuse treatment yet remain at high risk. In this case, conservative management was pursued based on preserved perfusion, systemic stability, and normal inflammatory markers. Post-transfer outcomes remain unknown, and caution is warranted in generalizing these findings to other patients. Recognition of atypical high-risk behaviours and the adoption of individualized, patient-centred strategies are critical to optimizing outcomes and mitigating further harm.
